# Identification of a Killer Toxin from *Wickerhamomyces anomalus* with β-Glucanase Activity

**DOI:** 10.3390/toxins11100568

**Published:** 2019-09-28

**Authors:** Valentina Cecarini, Massimiliano Cuccioloni, Laura Bonfili, Massimo Ricciutelli, Matteo Valzano, Alessia Cappelli, Consuelo Amantini, Guido Favia, Anna Maria Eleuteri, Mauro Angeletti, Irene Ricci

**Affiliations:** 1 School of Biosciences and Veterinary Medicine, University of Camerino, 62032 Camerino, Italy; 2 HPLC-MS Laboratory, University of Camerino, 62032 Camerino, Italy

**Keywords:** *Wickerhamomyces anomalus*, killer toxin, malaria, symbiotic control

## Abstract

The yeast *Wickerhamomyces anomalus* has several applications in the food industry due to its antimicrobial potential and wide range of biotechnological properties. In particular, a specific strain of *Wickerhamomyces anomalus* isolated from the malaria mosquito *Anopheles stephensi*, namely *Wa*F17.12, was reported to secrete a killer toxin with strong anti-plasmodial effect on different developmental stages of *Plasmodium berghei*; therefore, we propose its use in the symbiotic control of malaria. In this study, we focused on the identification/characterization of the protein toxin responsible for the observed antimicrobial activity of the yeast. For this purpose, the culture medium of the killer yeast strain *Wa*F17.12 was processed by means of lateral flow filtration, anion exchange and gel filtration chromatography, immunometric methods, and eventually analyzed by liquid chromatography-tandem mass spectrometry (LC–MS/MS). Based on this concerted approach, we identified a protein with a molecular weight of approximately 140 kDa and limited electrophoretic mobility, corresponding to a high molecular weight β-glucosidase, as confirmed by activity tests in the presence of specific inhibitors.

## 1. Introduction

Killer yeasts can secrete one or several kinds of toxins with peculiar molecular weights and post-translational modifications, which are able to kill sensitive cells of the same or related yeast genera in the absence of a direct cell-cell contact [1]. Killer toxin producers are immune to their own toxin but they can be susceptible to the toxins secreted by other killer yeasts [2]. Generally, these toxins act by targeting cell wall receptors of the sensitive cells with the killing mechanism consisting of a strong β-1,3-glucanase activity [3,4]. Toxin-producing yeasts are attracting great interest due to their several possible applications. In fact, they have been used as bio-control agents in food and fermentation industries to counteract possible contamination in the bio-typing of medically important pathogenic yeasts and fungi, as anti-fungal agents in the treatment of human and animal infections, and, recently, in the field of recombinant DNA technology [2,5,6,7].

The *Wickerhamomyces anomalus* is a *Saccharomyces* yeast with a wide spectrum of antimicrobial activities commonly used in food bio-preservation [7]. This yeast is observed in different habitats because it can adapt to a broad range of growth conditions in terms of osmolarity, temperature, and pH range values, showing tolerance to several environmental stress factors [7]. Interestingly, it stably colonizes pre-adult and adult stages of *Anopheles stephensi*, a primary vector of malaria in Asia. In particular, it localizes in the midgut and reproductive systems of both male and female mosquitoes, suggesting multiple transmission patterns [8]. Cappelli et al. reported that a specific strain of this yeast isolated from the mosquito *Anopheles stephensi*, namely *Wa*F17.12, releases a molecule that is recognized by an antibody specific for yeast killer toxins and that possesses antimicrobial activity against sensitive yeast strains, thus referring to it as a killer yeast [9]. Recently, in vitro and in vivo studies demonstrated that the same protein strongly affects different developmental stages of the murine malaria parasite *Plasmodium berghei*, identifying a β-glucanase-mediated mechanism of action and suggesting possible applications as a natural tool in the symbiotic control of malaria [10,11]. Considering these interesting properties, in this work, the secreted protein fraction of *Wa*F17.12 was first enriched by lateral flow filtration, separated by anion exchange and gel filtration chromatography, and the killer toxin of interest was finally characterized by immunometric methods and analyzed by reversed-phase liquid chromatography-tandem mass spectrometry (LC–MS/MS). Moreover, activity tests on sensible yeast cells were performed.

## 2. Results

### 2.1. Purification of the WaF17.12 Killer Toxin

**Figure 1:**
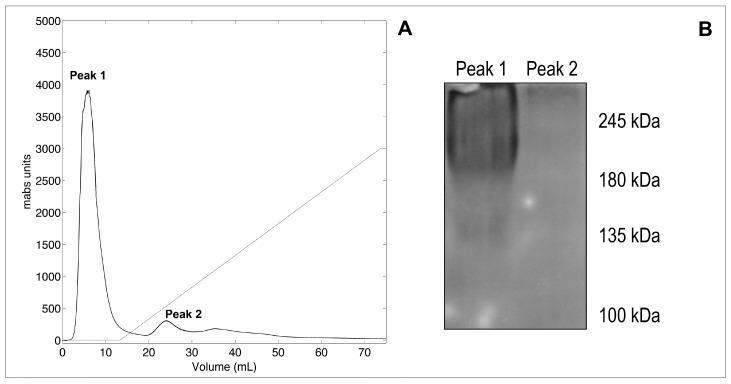
(**A**): elution profile obtained from the anion-exchange chromatography (DEAE) performed on the concentrated broth of the yeast *Wa*F17.12. (**B**): immunodetection of the *W. anomalus* KT on peak 1 and peak 2 with the mAbKT4 antibody.

The killer yeast strain *Wa*F17.12 (5 L working volume) was grown for 36 h at 26 °C and 70 shakes per min. The medium was then filtered and concentrated to a final volume of approximately 3 mL. The first step in the purification procedure consisted of a DEAE FF anion exchange chromatography that revealed the presence of a major peak corresponding to the unbound fraction that positively reacted with the monoclonal antibody mAbKT4 produced against a KT of the yeast W. *anomalus* ATCC 96603 [12] and gave a wide smeared electrophoretic band in the range of 130–250 kDa. Representative elution profile and western blotting results are shown in Figure 1. A killing activity test performed against the susceptible *Wa*UM3 strain further confirmed the presence of the KT in the unbound fraction of *Wa*F17.12 (data not shown).

**Figure 2:**
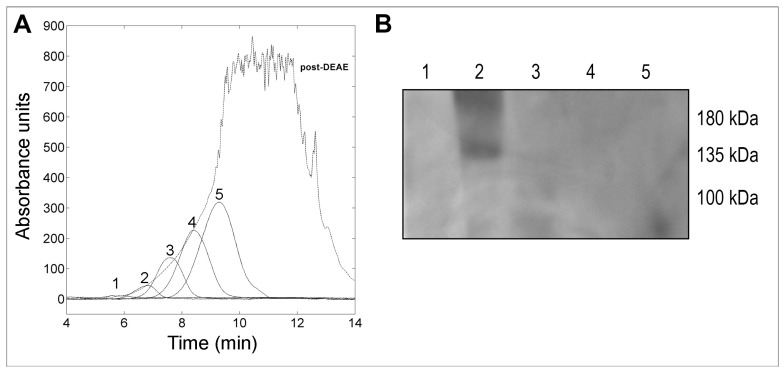
(**A**): superimposition of the profile obtained from the gel filtration chromatography (post-DEAE) with fractions obtained from independent analyses (peaks 1–5). (**B**): immunodetection of the *W. anomalus* KT in peaks 1–5 obtained after gel filtration using the mAbKT4 antibody.

The active fraction was dialyzed and further characterized by high-performance gel filtration chromatography using a progel-TSK G2000 SWXL column. The obtained elution profile is shown in Figure 2A and indicated as post-DEAE. Single fractions from multiple replicates were collected, combined and, subsequently, rerun to optimize the separation yield, obtaining the peaks 1–5 (Figure 2A). Individual peaks were extensively dialyzed and concentrated to a final volume of 500 µL. Western blotting assays performed with the obtained samples revealed the presence of the KT in peak 2, corresponding to a molecular weight of approximately 137 kDa, as shown in Figure 2B and Figure S1.

### 2.2. Identification of the Protein Toxin with Yeast Killing Activity

Tryptic peptides obtained upon digestion of peak 2 were studied by LC–MS/MS as described in the Materials and Methods. The analysis of resulting raw data with Mascot allowed the identification of five major peptide fragments (Table 1 and Figures S2–S6) covering 12% of a high-molecular weight β-glucosidase (EC: 3.2.1.21), with the primary sequence inferred from homology in four different strains of *W. anomalus* (UniProt entries: BGLS_WICAO, P06835, AFN27527.1, XP_019942137). This enzyme presents a conserved aspartic acid residue (D299) previously demonstrated to be implicated in its catalytic mechanism [13] and 18 sites of non-obligatory N-linked glycosylation (predicted using NetNGlyc 1.0 (http://www.cbs.dtu.dk/services/NetNGlyc/)).

### 2.3. Evaluation of KT Activity

**Figure 3:**
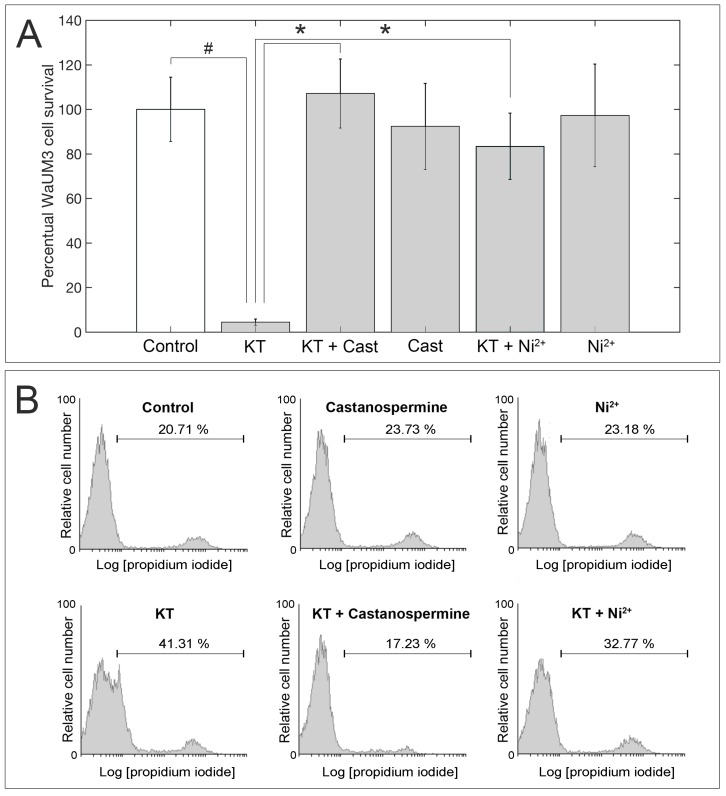
Killing activity of the isolated β-glucanase against the susceptible *Wa*UM3 strain. The addition of castanospermine and Ni^2+^, two β-glucanase inhibitors, strongly affects the activity of the *Wa*F17.12 killer protein. (**A**) shows data on *Wa*UM3 cells viability upon 12 h treatments. Values represent the mean ± S.D. of results obtained from three separate experiments. # indicates *p* < 0.01 compared to control and * indicates *p* < 0.01 compared to KT. (**B**) shows data obtained from flow cytometry analysis of treated *Wa*UM3 cells stained with PI. Percentages indicate PI-positive cells. Data shown are representative of three separate experiments.

To assess the β-glucanase activity of the identified killer protein, assays in the presence and in the absence of two specific inhibitors, castanospermine and Ni^2+^, were performed on the KT-sensitive strain *Wa*UM3. Yeast cells were grown on a 96-wells plate and incubated with PBS, the isolated protein, the isolated protein incubated with either castanospermine or Ni^2+^, and the individual inhibitors, respectively. The treatment with *Wa*F17.12-KT strongly affected *Wa*UM3 viability inducing a 90% decrease in yeast cell number (*p* < 0.01) compared to control (PBS) (Figure 3A). Interestingly, the activity of the toxin was blocked upon pre-incubation with either castanospermine or Ni^2+^, further confirming the β-glucanase-mediated mechanism of action of this killer protein. No effect on yeast viability was detected when cells were incubated only with the inhibitors (Figure 3A). Additionally, FACS analysis was performed in treated yeasts by staining with propidium iodide (PI), a dye that is able to intercalate double-strand DNA in cells with damaged membranes, allowing the detection of dying or dead cells. The increase in the percentage of PI-positive cells further corroborated the ability of the isolated *Wa*F17.12-KT to kill *Wa*UM3 cells according to a glucanase-mediated mechanism because the addition of both inhibitors markedly blocked its killer action (Figure 3B).

### 2.4. Docking Analysis of Castanospermine to W. anomalus β-Glucanase

**Figure 4:**
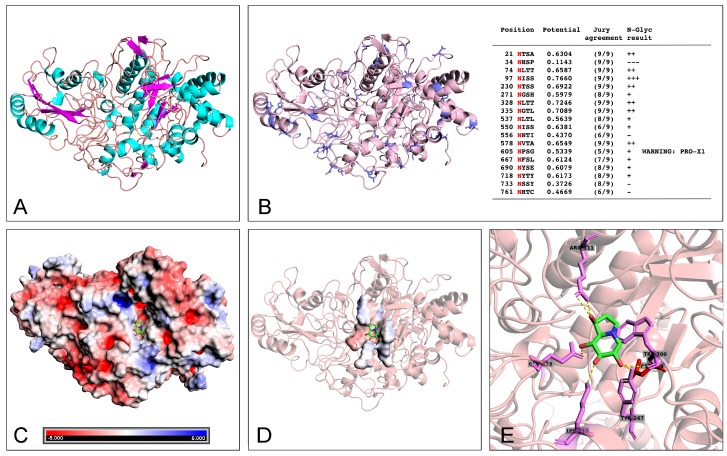
Three-dimensional representation of the structure of the β-glucanase isolated from *W. anomalus* F17.12 obtained by folding-assisted modeling and of the predicted complex thereof with castanospermine. Secondary structures of the enzyme are visualized in (**A**) (α-helices, light blue; β-sheets, violet), and Asn glycosylation sites are shown as solid blue sticks and summarized in the table inset of (**B**). Global and catalytic site local electrostatic potential surfaces (calculated with the Adaptive Poisson–Boltzmann Solver Tool—PyMol) are presented in (**C**) and (**D**), respectively. (**E**) close-up of the residues of the catalytic pocket involved in the interaction with castanospermine: catalytic Asp-299 is shown as a solid red stick, and all other residues predicted to form H-bonds with castanospermine (Arg-111, Lys-216, Tyr-267, Trp300, and Glu-523) are shown as solid magenta sticks. All images were rendered with PyMOL.

Docking studies between the fold-recognition model of *W. anomalus* β-glucanase (see Experimental Section for details) and the three-dimensional structure of castanospermine (PubChem CID: 54445) provided structural insights into the mechanism of action of the small molecule inhibitor. In agreement with activity tests, castanospermine was calculated to target the globular core of *W. anomalus* β-glucanase and accommodate in close proximity to the catalytic Asp-299. The resulting complex is mainly stabilized by the formation of six theoretical H-bonds with Arg-111, Lys-216, Tyr-267, Trp300, and Glu-523 (mean bond length: 2.6 Å). Three-dimensional representations of the protein and of the enzyme-inhibitor model are depicted in Figure 4D,E.

Predictive global energy, and individual contribution to the stabilization of the complex are summarized in Table 2.

## 3. Discussion

Killer toxins produced and released by yeasts are (glyco-) proteins that find numerous applications in pharmaceutical and food industries, and in biotechnology sector, making them interesting research targets [14]. Most killer toxins exert their activity on sensitive cells via a two-step mechanism: they bind to primary receptors, mainly β-glucans, on the cell wall of target cells and then are translocated to secondary receptors on the plasma membrane causing osmotic lysis and resulting in cell death [15]. 

The yeast *W. anomalous* produces several KTs, each characterized by variable molecular weights, a large range of optimal pH and temperature and a wide antimicrobial activity against several microorganisms [16,17]. The *Wa*F1.712 strain was previously isolated from the malaria vector *A. stephensi* and was shown to release a protein molecule that targets cell wall glucans with killer activity against other sensitive yeast strains/species and which is recognized by a monoclonal antibody specific for killer yeast toxins [9,10]. This toxin showed also a strong in vitro and in vivo anti-plasmodial activity against different developmental stages of the malaria parasite *P. berghei*, thus representing a promising tool for malaria control [9,11]. 

The goal of the present work was to definitely identify the protein responsible for this killing activity and to this aim, we implemented a rapid two-stage protocol, which consisted of the method previously described by Feng-Jun et al., with some modifications [17]. The toxin was isolated according to sequential anion exchange and gel filtration chromatography, the presence of the KT being always assessed with activity tests on sensitive yeasts and immune-assays using a specific primary antibody after each step. Generally, the fractions positive for the presence of the KT showed a typical electrophoretic pattern, consisting of a broad smear rather than a distinct band. In addition, the protein exhibited poor electrophoretic mobility with an apparent MW ranging approximately between 140 and 250 kDa, likely depending upon the variable degree of glycosylation of the protein [18,19]. 

The *Wa*F17.12 killer toxin was then definitely identified by LC–MS/MS analysis through the detection of five peptides. This large protein has a minimal molecular weight of 90 kDa (in the non-glycosylated form), in good agreement with the observed electrophoretic mobility (considering the presence of up to 18 non-obligatory glycosylation sites—Figure 4B). Activity assays in the presence of specific glucanase inhibitors confirmed our previously reported data on the ability of this killer protein to act on cell wall glucans [10].

In the absence of crystallographic data, structural information on the toxin and toxin-inhibitor complex were inferred from computational studies. Specifically, we derived a three-dimensional predictive model of *W. anomalus* KT consisting of a major globular core, with 32% helices (28% α-helices, 4% 3(10)-helices) and 12% β-sheets content (Stride Web Tool [20], Figure 4A and Figure S7), and extensive polar surfaces (Figure 4C). In particular, these surfaces are likely to play a key role during the enzyme-inhibitor (or substrate) recognition process, with specific regard to its positioning within the catalytic cleft of the enzyme (hydroxyl- and amino-groups of castanospermine are oriented toward the polar surfaces of the enzyme; Figure 4D). In fact, docking analysis predicted the inhibitor to accommodate in close proximity to the active site and hinder catalytic Asp-299, consistently with the competitive nature of castanospermine (Figure 4E) [21]. Additionally, besides being in apparent contrast to the results of electrophoretic analyses, the extent of polar surface implied a dramatic effect of the glycosyl modification on the electrophoretic mobility of the protein. 

In conclusion, the identification of this KT could open novel opportunities in the control of malaria and other mosquito-borne diseases, with the interesting prospect of using genetic engineering techniques to opportunely exploit its killer activity as a tool to effectively reduce the burden of such disorders. 

## 4. Materials and Methods 

### 4.1. Materials

Salts were purchased from Sigma-Aldrich (Milan, Italy). Reagents were obtained from Mallinckrodt Baker (Milan, Italy). The Sartorius Vivaflow 200 ultrafiltration system, equipped with a 10 kDa cutoff polythersulfone membrane, was obtained from Sartorius (Florence, Italy). Pierce Concentrators were from Thermo Fisher Scientific (Milan, Italy). Anion exchange chromatography was performed on an FPLC Akta Basic equipped with a UV-Vis detector (GE-Healthcare, Milan, Italy) using a HiTrap DEAE FF column with a volume of 5 mL (GE-Healthcare, Milan, Italy). Gel filtration chromatography was conducted on an HPLC Akta Basic equipped with a UV-Vis detector (GE Healthcare, Milan, Italy) using a progel-TSK G2000 SWXL column 30 cm × 7.8 mm (Supelco, Merck KGaA, Darmstadt, Germany). Dialysis was performed using Spectra/Por 3.5 kDa MWCO membranes (Spectrum Labs, Fisher Scientific Italia, Rodano, Milan). PVDF membranes for western blotting were obtained from Millipore (Milan, Italy). The monoclonal antibody mAbKT4 was used to detect the KT [9]. The anti-mouse secondary antibody was purchased from Santa Cruz Biotechnology (Heidelberg, Germany). The ECL (enhanced chemiluminescence) system used for the immunodetection was obtained from Amersham Pharmacia Biotech (Milan, Italy). 

### 4.2. Yeast Strains and KT Production

The *W. anomalus* strain *Wa*F17.12 was isolated from *A. stephensi* mosquitoes [8]. The yeast was grown in YPD liquid medium (20 g/L peptone, 20 g/L glucose, 10 g/L yeast extract) buffered at pH 4.5 with 0.1 M citric acid and 0.2 M K_2_HPO_4_ and incubated at 26 °C for 36 h at 70 shakes per min, as reported elsewhere [9,22]. The KT-susceptible and non-producing strain *Wa*UM3 was grown under the same conditions and was used in activity assays to evaluate the presence of KT in the chromatographic fractions [9].

### 4.3. Sample Processing and Concentration

Yeast processing conditions were optimized, changing the initial volume of yeast medium (in the range 0.5–-5 L) and the concentration factor (100–-1000×) to obtain a sufficient amount of killer protein for all the subsequent evaluations. The yeast culture (5 L) obtained after 36 h incubation was centrifuged at 4,000 rpm for 10 min (T = 4 °C) to remove yeast cells. The supernatant was filtered using 0.22 μm sterile filtering flasks (Millipore, Billerica, MA, USA) and concentrated 100× using the Sartorius Vivaflow 200 ultrafiltration system equipped with a polyethersulfone membrane (cutoff: 10 kDa). The obtained volume was further reduced (10×) using concentrators tube (cutoff: 10 kDa) centrifuged at 6,000 rpm for 1 h at 4 °C. 

### 4.4. Anion Exchange Chromatography

The resulting solution was processed by anion-exchange chromatography on an FPLC AKTA Basic device equipped with a HiTrap DEAE FF column equilibrated with 0.1 M citric acid and 0.1 M Na_2_HPO_4_ buffer, pH 4.5. Elution of the bound species was performed using a linear gradient of the same buffer added with 1 M NaCl (flow rate 5 mL/min). Retained and non-retained fractions were collected from different runs, combined and quantified for protein content according to the method of Bradford, using bovine serum albumin as standard [23]. The presence of the KT was evaluated by western blotting assays using the monoclonal antibody mAbKT4 produced against a KT of the *W. anomalus* ATCC 96603 [12] and by activity assays against the susceptible strain *Wa*UM3.

### 4.5. Gel Filtration Chromatography

The fraction containing the KT was applied to a progel-TSK G2000 SWXL column and components were separated by isocratic elution with 0.1 M Na_2_SO_4_ and 0.1 M Na_2_HPO_4_, pH 6.7, flow rate 1 mL/min. Corresponding fractions resulting from different runs were collected, combined and, subsequently, were rerun to optimize the separation. A mixture of molecular weight markers (Bio-Rad lab) was run to estimate the weight of the killer toxin. Single fractions were dialyzed, lyophilized, and then tested for the presence of the toxin by Western blotting. 

### 4.6. Western Blotting

Fractions obtained after the anion exchange and gel filtration chromatography were tested for the presence of the toxin through Western blotting analyses. Protein content was determined by the Bradford method [23]. Proteins were separated on a 7% polyacrylamide gel and then electrically transferred to a polyvinylidene difluoride (PVDF) membrane. Membranes with transferred proteins were incubated with the primary monoclonal antibody KT4 produced against a KT of *W. anomalus* ATCC 96603 and then with an anti-mouse secondary antibody. Protein detection was performed with an ECL Western blotting analysis system [24]. Each gel was loaded with pre-stained molecular weight markers in the range 10–245 kDa (Nippon Genetics, Düren, Germany).

### 4.7. Sample Preparation for LC−MS/MS

The conditions for protein reduction, alkylation, and digestion were essentially those reported by Geisslitz et al., with minor changes [25]. Briefly, the protein fraction with a molecular weight in the range 100–200 kDa (Vol: 100 μL, 50 μg/mL in 0.5 M Tris-HCl, pH) was reduced by the addition of 1,4-dithiothreitol (DTT) (Vol: 50 μL, 0.05 M in 0.5 M Tris-HCl, pH 8.5) and incubated for 30 min at 60 °C. Cysteine residues were alkylated with iodoacetamide (Vol: 50 μL, 0.5 M in 0.5 M Tris-HCl, pH 8.5) for 45 min at 37 °C in the dark, then freeze-dried. Tryptic hydrolysis (0.5 mL, enzyme-to-protein ratio 1:50, 0.04 M urea in 0.1 M Tris-HCl, pH 7.8) was performed for 24 h at 37 °C in the dark. The reaction was stopped with 2 μL of trifluoroacetic acid. A final clean-up step was performed with 1 mL Sep-Pak C18 cartridges (Waters, Milford, MA, USA). The cartridges were pre-conditioned using 1 mL acetonitrile, and equilibrated with a solution of 80 % CH_3_CN, 0.1 % TFA. The trypsin digest was loaded and washed with a solution of 5 % CH_3_CN, 0.1 % TFA. The flow-through and the wash were collected and processed by a second SPE cleanup procedure to recover peptides not retained during the first SPE step. Elution was done with 80% CH_3_CN, 0.1% HCOOH. The eluates were freeze-dried under vacuum and stored at −20 °C until use.

### 4.8. LC–MS/MS Analysis

Tryptic digests were dissolved in 100 μL of 0.1% (v/v) trifluoroacetic acid, vortexed and incubated for 5 min in a sonication bath and centrifuged for 15 min at 10000 rpm. Each sample was analyzed in LC-ESI/MS using an HPLC 1100 Series (Agilent Technologies, Santa Clara, CA, USA) coupled to an Ion Trap Mass Spectrometer (Agilent Technologies LC/MSD Trap SL) with an electrospray ion source (ESI) operating in positive ion mode over the mass range 300–2200 amu (atomic mass units). MS spray voltage was 3.5 kV and the capillary temperature was maintained at 300 °C. The column was a reversed-phase C18 Gemini-NX, 5 µm particle size, 110 Å pore size, 250x4.6 mm, (Phenomenex, Torrance, CA, USA). The injection volume was set at 80 µL and the temperature of the column was set at 40 °C. The elution was done with a 45 min linear gradient from 90:10 A:B to 10:90 A:B (A: water 0.1% formic acid; B: acetonitrile 0.1% formic acid).

Mass spectra were analyzed with MASCOT software [26], with search parameters being set as follows: Database: NCBIprot; Taxonomy: Fungi; Enzyme: trypsin; Fixed modification: Carbamidomethyl (C); Variable modification: Carbamidomethyl (N-term), Oxidation (M); Peptide tolerance: ±1.2 Da; MS/MS tolerance: ±0.6 Da; One missed cleavage allowed.

### 4.9. Activity Assays 

An aliquot of the purified product was tested against the susceptible strain *Wa*UM3 in the presence of the β-glucanase inhibitors castanospermine and Ni^2+^ [10,27]. The fraction containing the killer protein was buffer-exchanged with a Hi-Trap desalting column to PBS 1× (10 mM Na_2_HPO_4_, 2.7 mM KCl, 138 mM NaCl, pH 7.4) to prevent possible interference, like pH-incompatibility, with the yeast cells viability. Cells were spotted in a 96-well microtiter plate (10^5^ cells/well) and then independently incubated with PBS, the isolated protein (100 µg/mL), the inhibitor (25 µM castanospermine or 10 µM Ni^2+^, in line with available data on their inhibitory potency [27,28]), and the isolated protein and the inhibitor, respectively. KT and inhibitors were pre-incubated for 1 h at 25 °C [29]. To assure the normal growth of the *Wa*UM3 strain, cells were grown in fresh YPD medium and free medium was spotted in additional wells to exclude possible contaminations. After 12 h incubation at 26 °C, the toxic activity was evaluated by checking cell morphology and analyzing the cellular viability through the trypan blue assay on a Neubauer counting chamber using an optical microscope with a 40× objective (Carl Zeiss Axio Observer.Z1, Milan, Italy). Assays were performed in triplicate. The data from the cell counts were analyzed using GraphPad Prism® 5.0a (GraphPad Software, La Jolla, CA, USA, www.graphpad.com) and statistical analysis was performed by multiple comparisons using the Mann–Whitney test. Statistical significance is expressed as a p-value (*p* < 0.01). 

In addition, after treatment, yeast cells were stained for 30 min at 20 °C with 20 µg/mL of the red-fluorescent (FL-2) dye propidium iodide (PI) (Sigma-Aldrich, Saint Louis, MO, USA) in DNAse-free PBS and finally analyzed by a FACScan flow cytometer (BD Biosciences, Milan, Italy) using the CellQuest software. The percentage of positive cells was determined over 10,000 events.

### 4.10. Prediction of Three-Dimensional Structures of WaF17.12 β-Glucanase

P06835 UniProt entry sequence was used to model the three-dimensional structure of the *β-glucanase* from *W. anomalus,* as reported by Cuccioloni et al. [30]. The signal peptide (MLLPLYGLASFLVLSQAALV) was predicted using SignalP [31] and the N-terminus sequence shortened consequently. Fold-recognition modeling was performed using I-Tasser [32], the best structural templates being 4iibA, 5nbsA, 4d0j, 5fjjA. Structure refinement of the predicted best scoring model was carried out using ModRefiner [33]. Energy minimization of the output model was performed with a GROMOS 96 forcefield [34] and the resulting predictive structure was submitted to PROCHECK for backbone structure validation [35].

### 4.11. β-Glucanase-Castanospermine Docking Analysis

The predictive models of the complex between *W. anomalus* β-glucanase and castanospermine (PubChem CID: 54445 [36]) was computed by docking the drug to the fold-recognition model of the protein. Rigid docking was performed using PatchDock server [37], *W. anomalus* β-glucanase and castanospermine being uploaded as receptor and ligand, respectively (Complex type: enzyme-inhibitor; Clustering RMSD: 4.0; No constraints applied on enzyme or inhibitor binding sites). FireDock [38] was used for interaction refinement. (Refinement levels: full; Number of RBO cycles: 50; Atomic Radius Scale: 0.8 Å; Receptor/Ligand: bound; Residues/bonds: flexible). The best scoring complex and all images were rendered with PyMOL (The PyMOL Molecular Graphics System, Version 1.3 Schrödinger, LLC, New York, NY, USA).

## Figures and Tables

**Table 1:** Main peptide ions associated to the β-glucanase protein of interest displayed in ESI-TRAP mass spectra.

Observed (M^2+^)	Mr (Expected)	Mr (Calculated)	Missed Cleavage	Peptide
753.39	1,504.7654	1,504.7555	1	ARELVDQMSIAEK + Oxidation (M)
987.02	1,972.0254	1,972.0200	1	GADAILGPVYGPMGVKAAGGR + Oxidation (M)
637.83	1,273.6454	1,273.6514	0	ISILGQAAGDDSK
1,088.02	2,174.0254	2,174.0386	0	VNLTTGVGSASGPCSGNTGSVPR
1,456.16	2,910.3054	2,910.3243	0	GCGSGAIGTGYGSGAGTFSYFVTPADGIGAR

**Table 2:** Predictive individual energy contribution to the stabilization of the complex between *W. anomalus* β-glucanase and castanospermine, expressed as kcal/mol (aVdW, rVdW: softened attractive and repulsive van der Waals energy, ACE—atomic contact energy; Inside—insideness measure).

Global Energy	aVdW	rVdW	ACE	Inside
−19.49	−11.04	1.14	−4.42	4.62

## Supplementary Materials

The following are available online at https://www.mdpi.com/2072-6651/11/10/568/s1, Figure S1: Superimposition of fractions obtained from gel filtration analyses and molecular weight markers, Figure S2: MS/MS spectrum of [M+2H]^2+^ at m/z = 753.39 assigned to peptide AA 59–72 of *W. anomalus* β-glucanase, Figure S3: MS/MS spectrum of [M+2H]^2+^ at m/z = 1088.02 assigned to peptide AA 73–95 of *W. anomalus* β-glucanase, Figure S4: MS/MS spectrum of [M+2H]^2+^ at m/z = 987.02 assigned to peptide AA 148–169 of *W. anomalus* β-glucanase, Figure S5: MS/MS spectrum of [M+2H]^2+^ at m/z = 637.83 assigned to peptide AA 433–445 of *W. anomalus* β-glucanase, Figure S6: MS/MS spectrum of [M+2H]^2+^ at m/z = 1456.16 assigned to peptide AA 453–483 of *W. anomalus* β-glucanase, Figure S7: Prediction of *W. anomalus* KT secondary structure.
