# Supplementary material for: Identification of a Killer Toxin from Wickerhamomyces anomalus with β-Glucanase Activity
Source: Toxins (Basel). 2019 Sep 28;11(10):568. doi: 10.3390/toxins11100568 (PMC6832412; doi:10.3390/toxins11100568)
Supplement: Supplementary file 1 [file toxins-11-00568-s001.pdf]

Supplementary Materials: Identification of a Killer Toxin from *Wickerhamomyces anomalus* with β-glucanase Activity

Valentina Cecarini, Massimiliano Cuccioloni, Laura Bonfili, Massimo Ricciutelli, Matteo Valzano, Alessia Cappelli, Consuelo Amantini, Guido Favia, Anna Maria Eleuteri, Mauro Angeletti and Irene Ricci

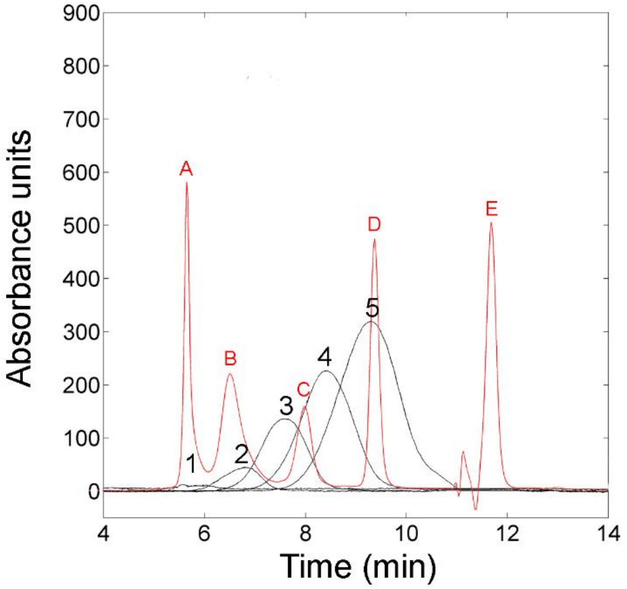

Figure S1. Superimposition of fractions obtained from gel filtration analyses and molecular weight markers (red chromatogram, A: Thyroglobulin 670 kDa, B: γ-globulin 158 kDa, C: Ovalbumin 44 kDa, D: Myoglobin 17 kDa, E: Vitamin B12 1.35 kDa).

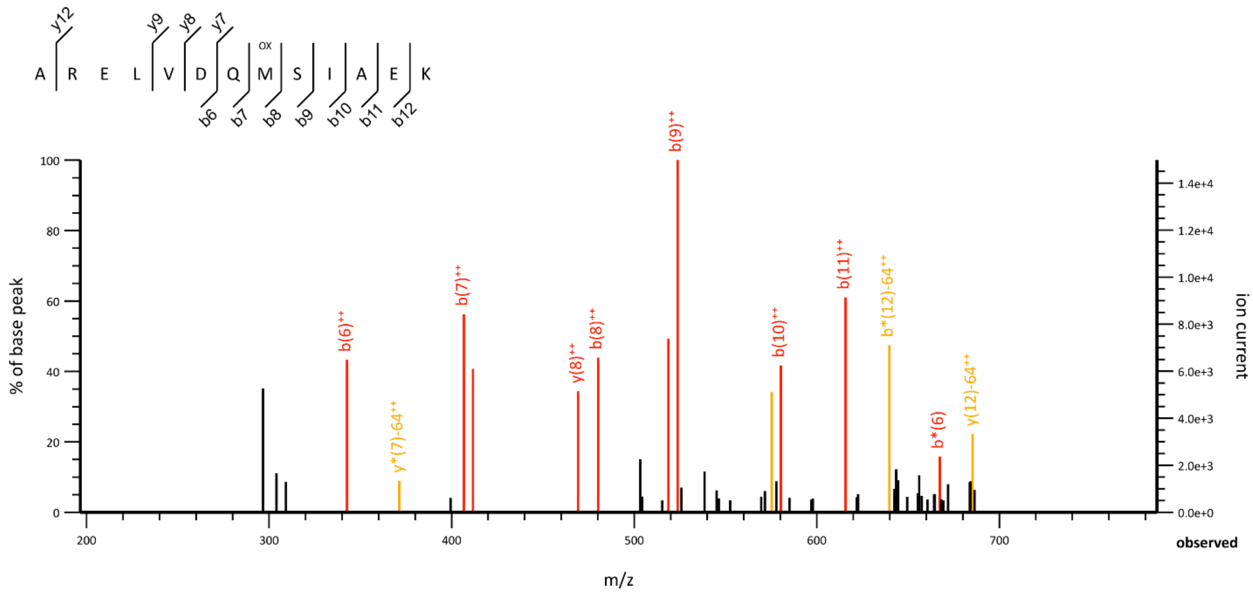

Figure S2. MS/MS spectrum of [M+2H]$^{2+}$ at m/z = 753.39 assigned to peptide AA 59–72 of *W. anomalus* β-glucanase. Ion series y and b were labeled according to Matrix Science—Peptide Fragmentation [http://www.matrix science.com/help/fragmentation_help.html].

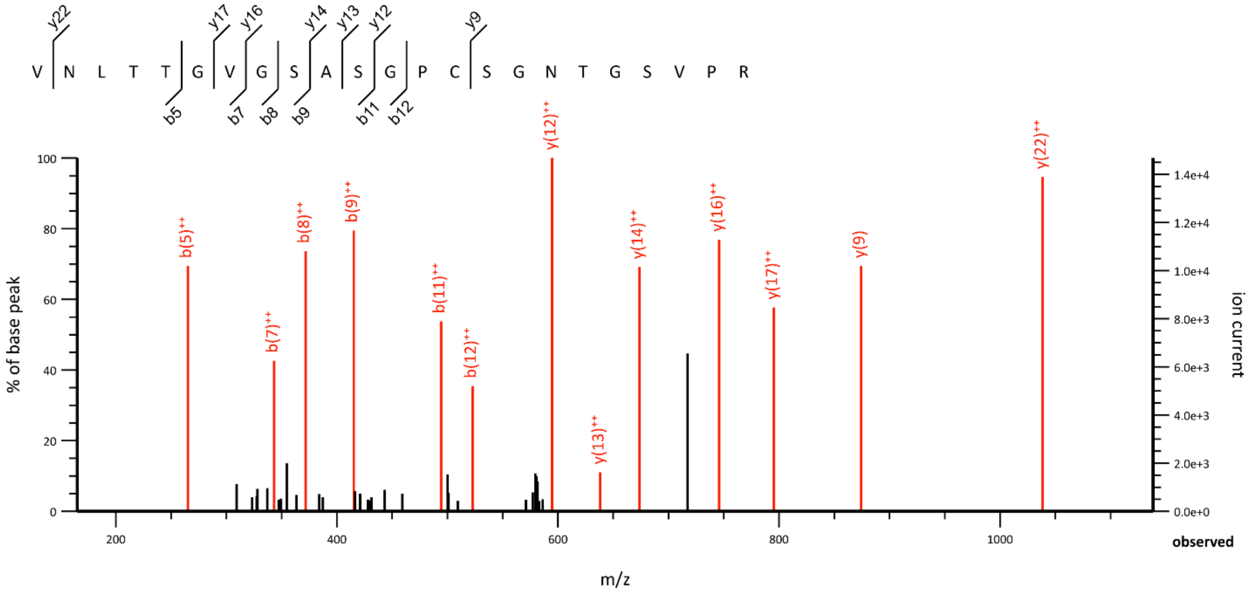

Figure S3. MS/MS spectrum of [M+2H]$^{2+}$ at m/z = 1088.02 assigned to peptide AA 73–95 of *W. anomalus* β-glucanase. Ion series y and b were labeled according to Matrix Science—Peptide Fragmentation [http://www.matrix science.com/help/fragmentation_help.html].

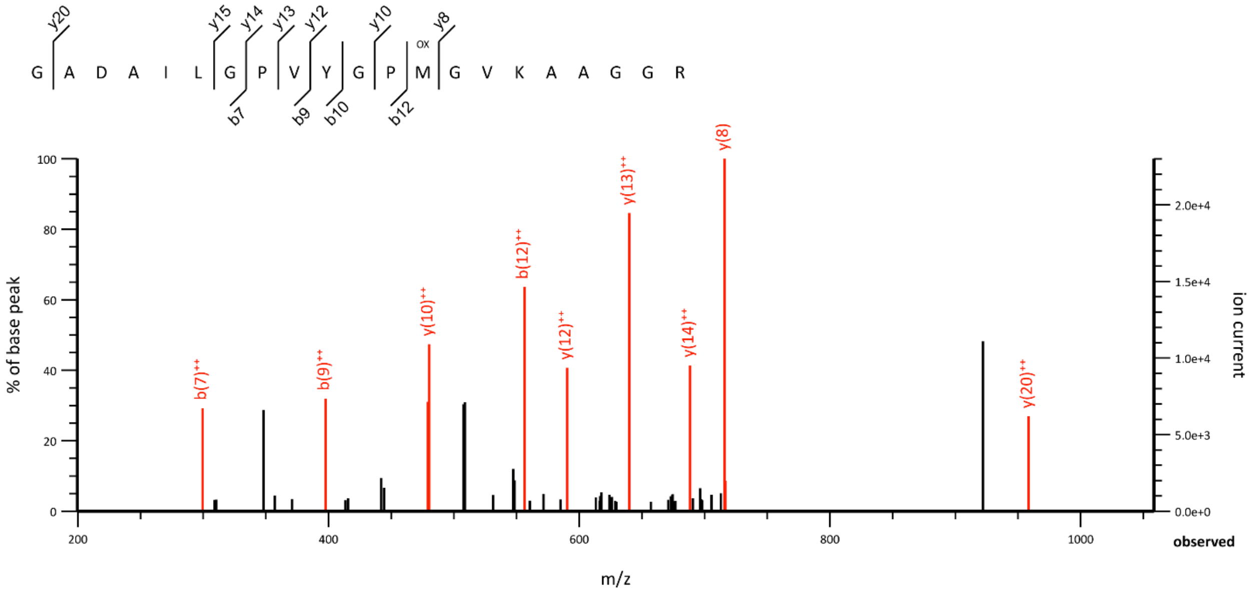

Figure S4. MS/MS spectrum of [M+2H]$^{2+}$ at m/z = 987.02 assigned to peptide AA 148–169 of *W. anomalus* β-glucanase. Ion series y and b were labeled according to Matrix Science—Peptide Fragmentation [http://www.matrix science.com/help/fragmentation_help.html].

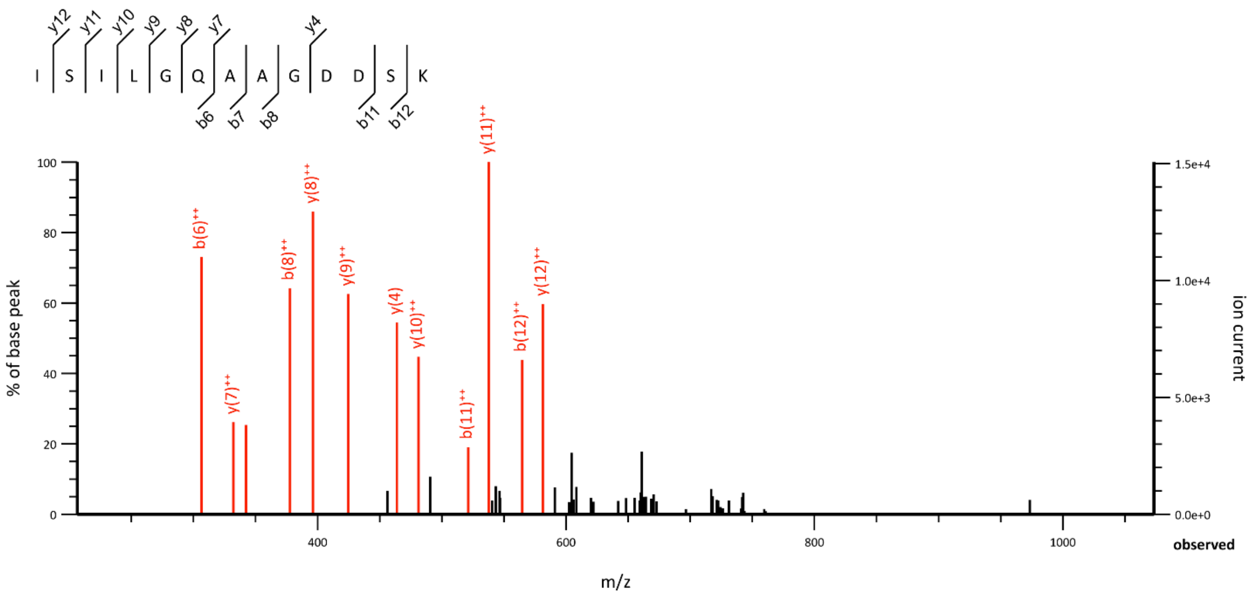

Figure S5. MS/MS spectrum of [M+2H]$^{2+}$ at m/z = 637.83 assigned to peptide AA 433–445 of *W. anomalus* β-glucanase. Ion series y and b were labeled according to Matrix Science—Peptide Fragmentation [http://www.matrix science.com/help/fragmentation_help.html].

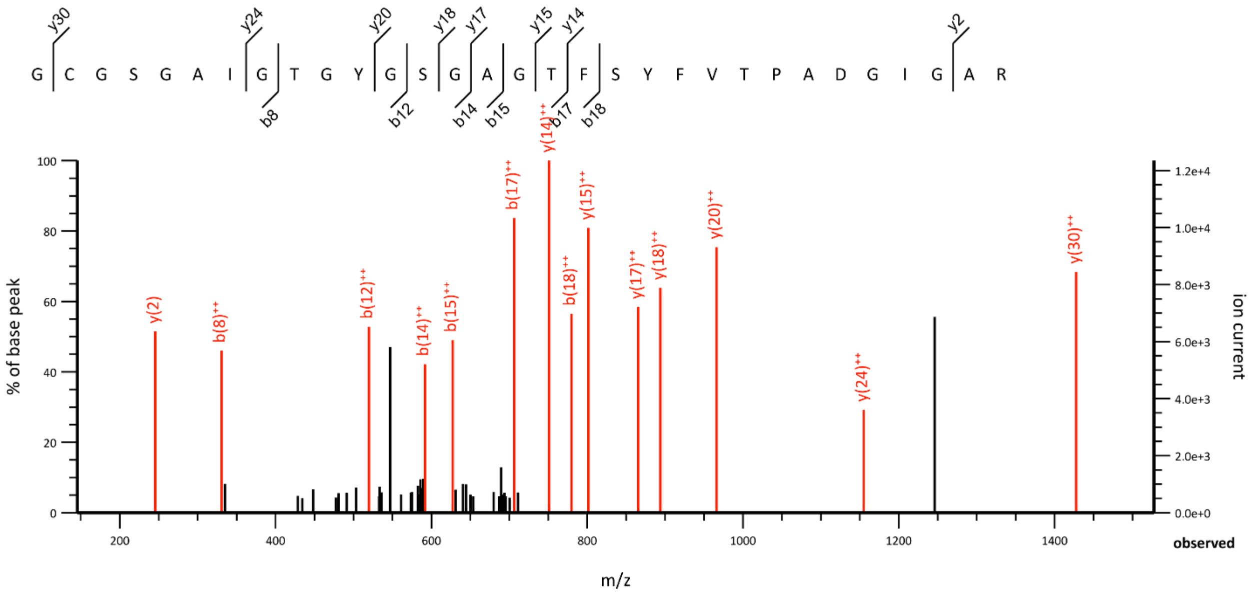

Figure S6. MS/MS spectrum of [M+2H]$^{2+}$ at m/z = 1456.16 assigned to peptide AA 453–483 of *W. anomalus* β-glucanase. Ion series y and b were labeled according to Matrix Science—Peptide Fragmentation [http://www.matrix science.com/help/fragmentation_help.html].

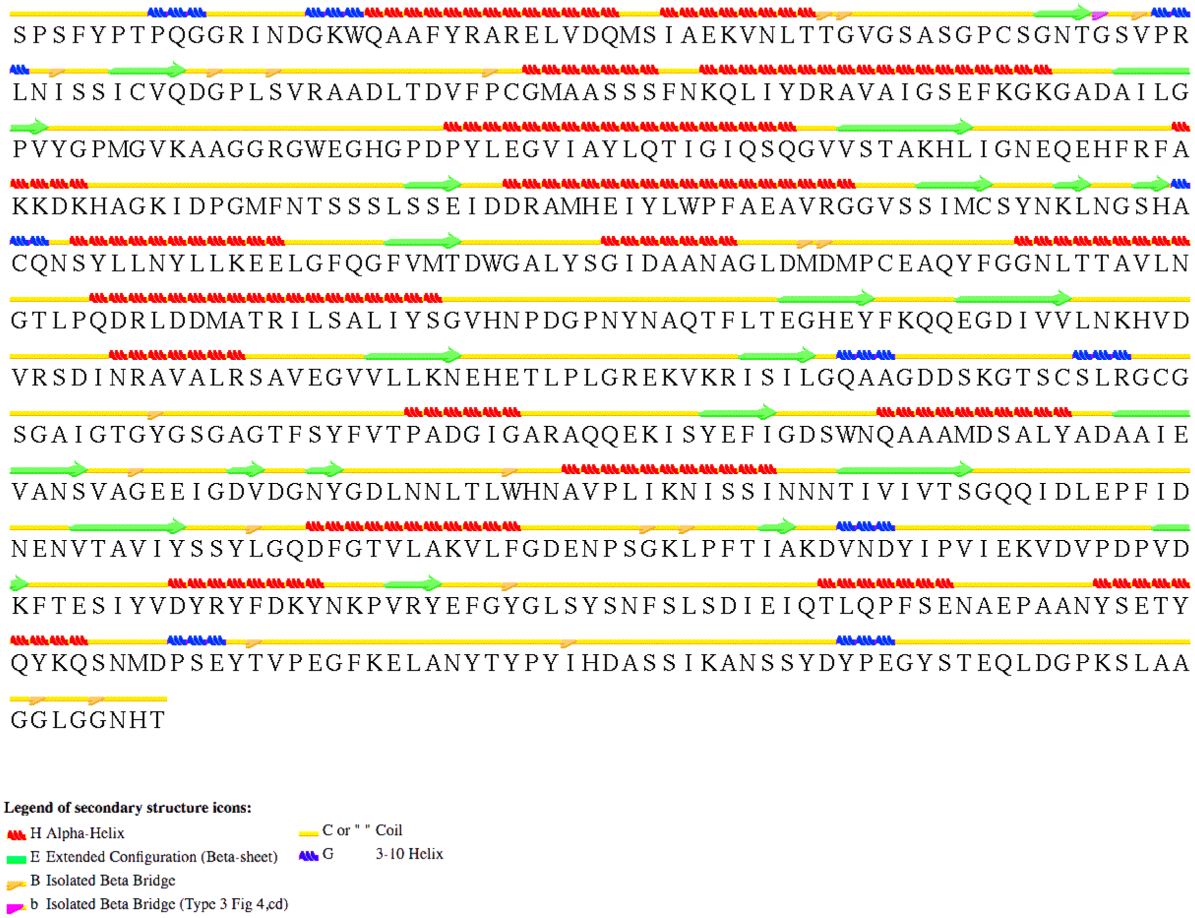

Figure S7. Prediction of *W. anomalus* KT secondary structure.
